# Supplementary material for: Bioactive Dimeric Abietanoid Peroxides from the Bark of Cryptomeria japonica
Source: Molecules. 2019 Jun 10;24(11):2178. doi: 10.3390/molecules24112178 (PMC6600475; doi:10.3390/molecules24112178)
Supplement: Supplementary file 1 [file molecules-24-02178-s001.pdf]

Supplementary Materials

Bioactive Dimeric Abietanoid Peroxides from the Bark of *Cryptomeria japonica*

Chi-I Chang [1,2], Cheng-Chi Chen [3,†], Chiy-Rong Chen [4], Ming-Der Wu [5], Ming-Jen Cheng [5], Ping-Jyun Sung [6,7,†] and Yueh-Hsiung Kuo [8,9,10,*]

[1] Department of Biological Science and Technology, National Pingtung University of Science and Technology, Pingtung 912, Taiwan; changchii@mail.npust.edu.tw
[2] Research Center for Active Natural Products Development, National Pingtung University of Science and Technology, Pingtung 912, Taiwan
[3] Department of Chemistry, National Taiwan University, Taipei 106, Taiwan; r93223083@ntu.edu.tw
[4] Department of Life Science, National Taitung University, Taitung 950, Taiwan; gina77@nttu.edu.tw
[5] Bioresource Collection and Research Center (BCRC), Food Industry Research and Development Institute (FIRDI), Hsinchu 300, Taiwan; wmd@firdi.org.tw (M.-D.W.); cmj@firdi.org.tw (M.-J.C.)
[6] National Museum of Marine Biology and Aquarium, Pingtung 944, Taiwan; pjsung@nmmba.gov.tw
[7] Graduate Institute of Marine Biology, National Dong Hwa University, Pingtung 944, Taiwan
[8] Department of Chinese Pharmaceutical Sciences and Chinese Medicine Resources, College of Pharmacy, China Medical University, Taichung 404, Taiwan
[9] Department of Biotechnology, Asia University, Taichung 413, Taiwan
[10] Chinese Medicine Research Center, China Medical University, Taichung 404, Taiwan
[*] Correspondence: kuoyh@mail.cmu.edu.tw; Tel.: +886-4-2205-3366 (ext. 5701); Fax: +886-4-2207-1693
[†] These two authors contributed equally to this paper.

CONTENT

Figure S1. ^{1}H-NMR spectrum of compound **1** in $CDCl_3$.
Figure S2. ^{13}C-NMR spectrum and DEPT of compound **1** in $CDCl_3$.
Figure S3. HMQC spectrum of compound **1** in $CDCl_3$.
Figure S4. HMBC spectrum of compound **1** in $CDCl_3$.
Figure S5. ^{1}H-^{1}H COSY spectrum of compound **1** in $CDCl_3$.
Figure S6. NOSEY spectrum of compound **1** in $CDCl_3$.
Figure S7. IR spectrum of compound **1**.
Figure S8. Mass spectrum of compound **1**.
Figure S9. UV-Vis spectrum of compound **1**.
Figure S10. ^{1}H-NMR spectrum of compound **2** in $CDCl_3$.
Figure S11. ^{13}C-NMR spectrum and DEPT of compound **2** in $CDCl_3$.
Figure S12. HMQC spectrum of compound **2** in $CDCl_3$.
Figure S13. HMBC spectrum of compound **2** in $CDCl_3$.
Figure S14. ^{1}H-^{1}H COSY spectrum of compound **2** in $CDCl_3$
Figure S15. NOSEY spectrum of compound **2** in $CDCl_3$.
Figure S16. IR spectrum of compound **2**.
Figure S17. Mass spectrum of compound **2**.
Figure S18. UV-Vis spectrum of compound **2**.
Figure S19. ^{1}H-NMR spectrum of compound **3** in $CDCl_3$.
Figure S20. ^{13}C-NMR spectrum and DEPT of compound **3** in $CDCl_3$.
Figure S21. HMQC spectrum of compound **3** in $CDCl_3$.
Figure S22. HMBC spectrum of compound **3** in $CDCl_3$.
Figure S23. ^{1}H-^{1}H COSY spectrum of compound **3** in $CDCl_3$.
Figure S24. NOSEY spectrum of compound **3** in $CDCl_3$.
Figure S25. IR spectrum of compound **3**.
Figure S26. Mass spectrum of compound **3**.
Figure S27. UV-Vis spectrum of compound **3**.
Figure S28. ^{1}H-NMR spectrum of compound **4** in $CDCl_3$.
Figure S29. ^{1}H-NMR spectrum of compound **5** in $CDCl_3$.
Figure S30. ^{1}H-NMR spectrum of compound **6** in $CDCl_3$.
Figure S31. ^{1}H-NMR spectrum of compound **7** in $CDCl_3$.
Figure S32. HPLC separation chromatogram of compounds **1** and **2**.

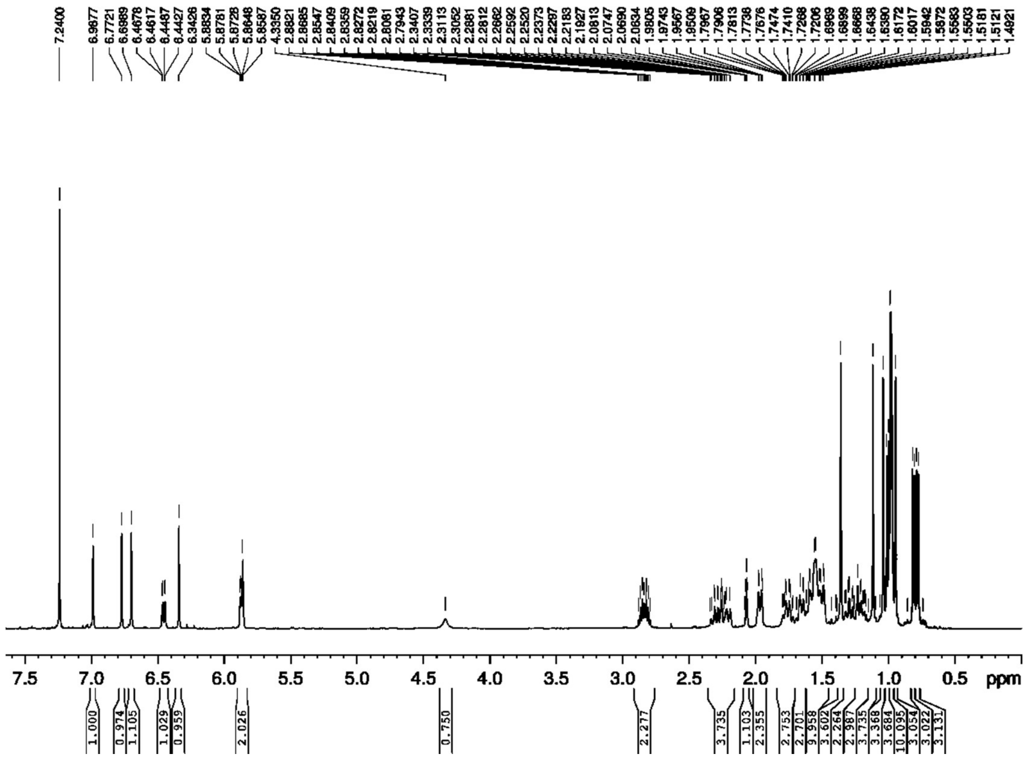

Figure S1. ^{1}H-NMR spectrum of compound **1** in $CDCl_3$.

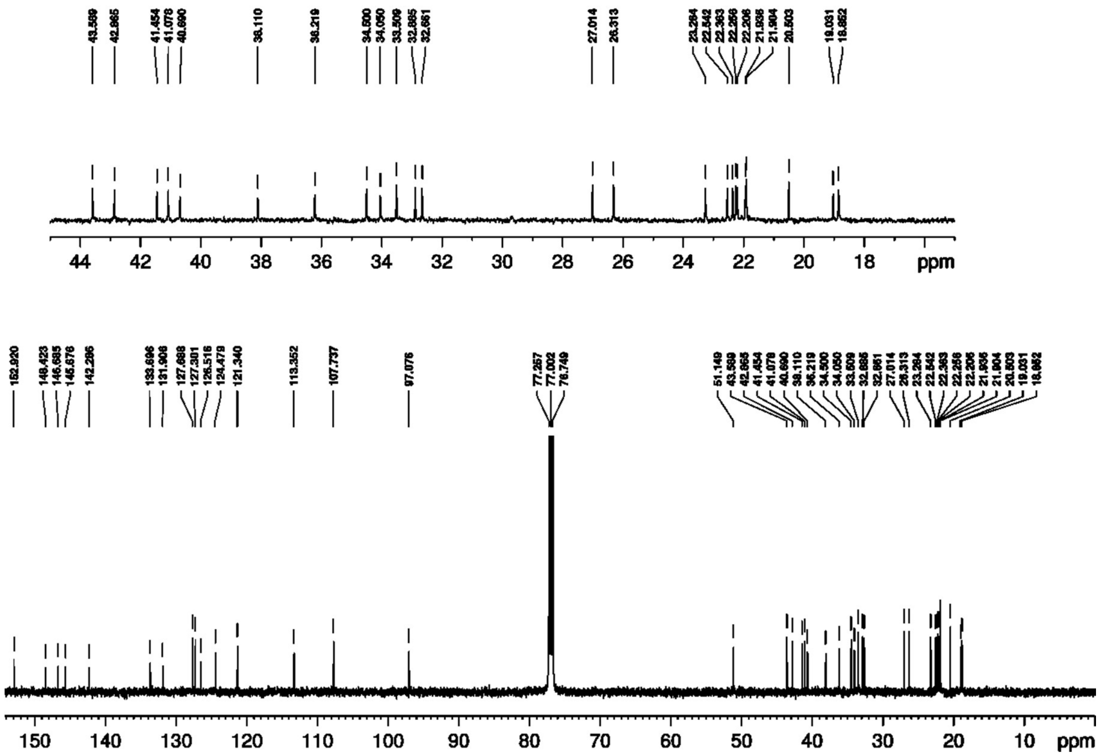

Figure S2. ^{13}C-NMR spectrum and DEPT of compound **1** in $CDCl_3$.

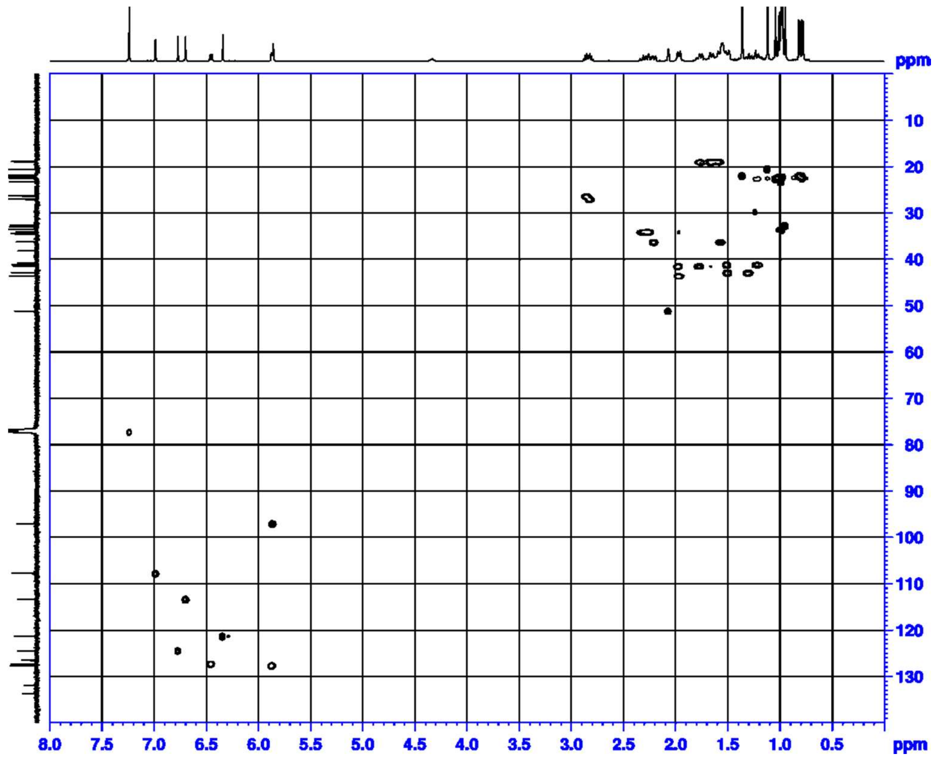

Figure S3. HMQC spectrum of compound **1** in $CDCl_3$.

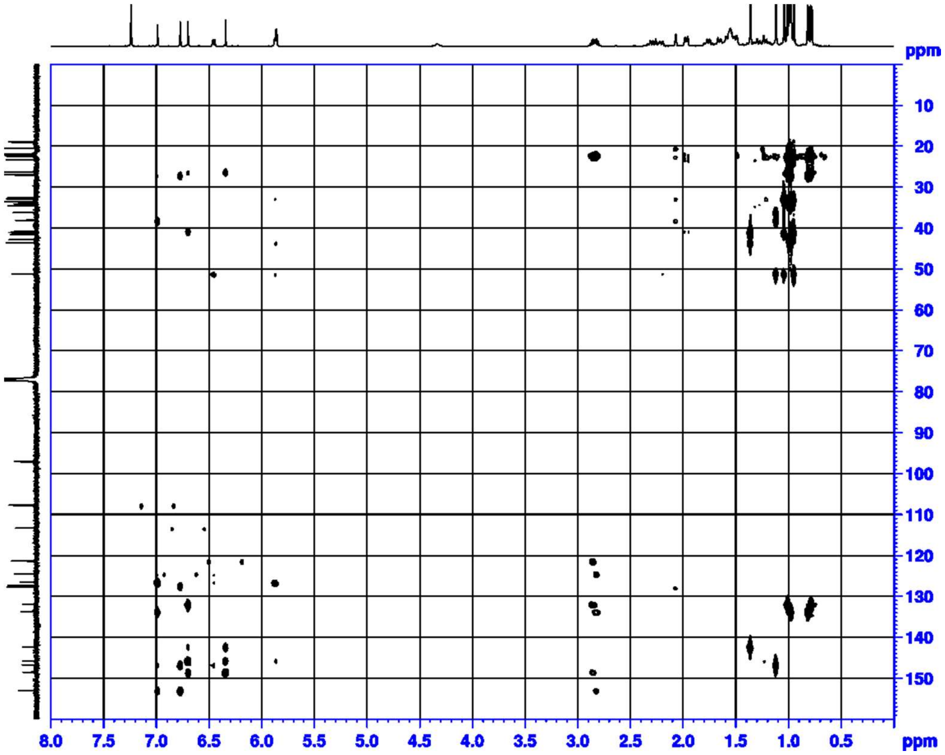

Figure S4. HMBC spectrum of compound **1** in $CDCl_3$.

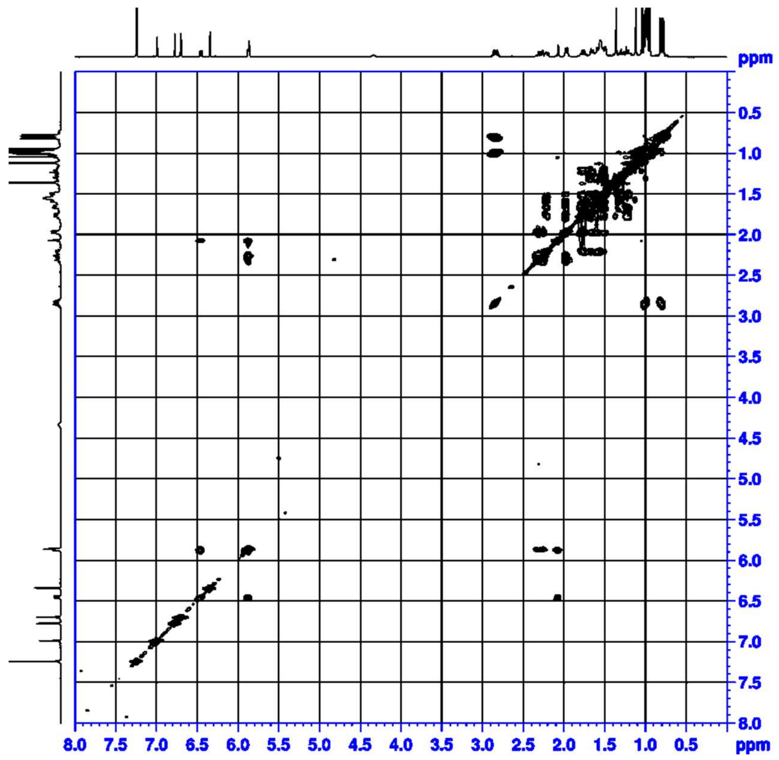

Figure S5. ^{1}H-^{1}H COSY spectrum of compound **1** in $CDCl_3$.

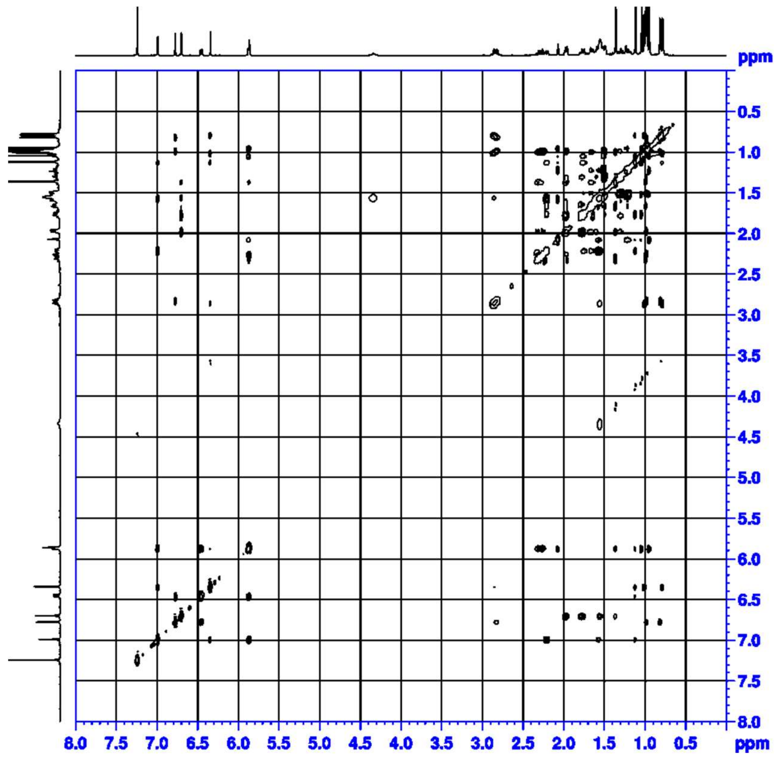

Figure S6. NOSEY spectrum of compound **1** in $CDCl_3$.

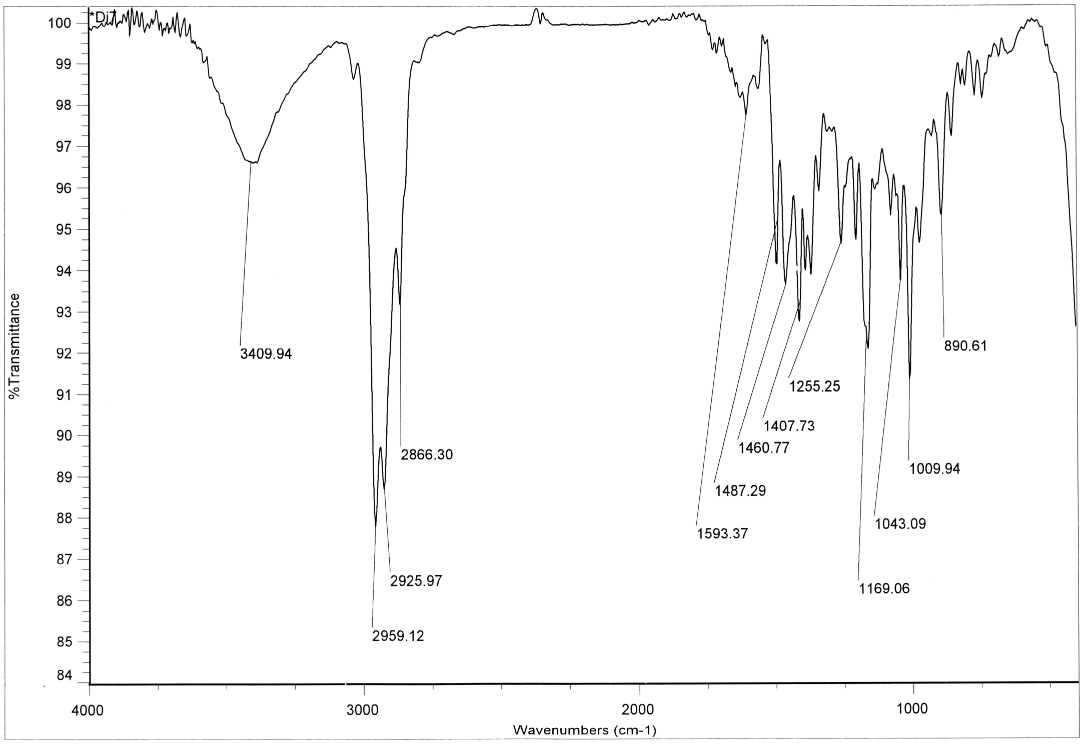

Figure S7. IR spectrum of compound **1**.

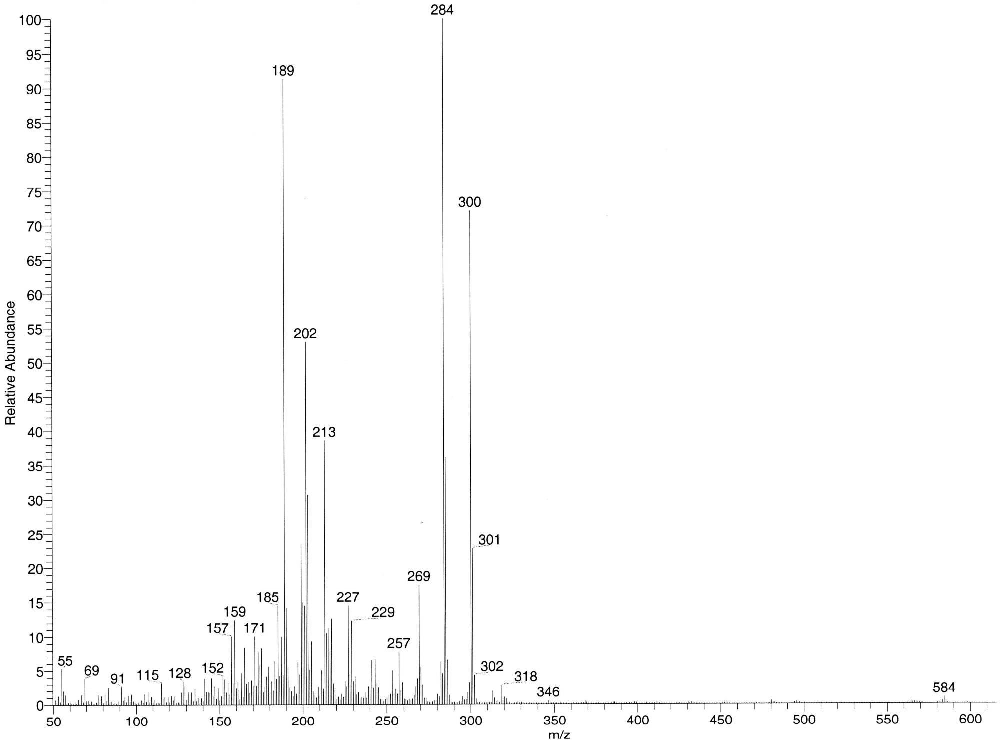

Figure S8. Mass spectrum of compound **1**.

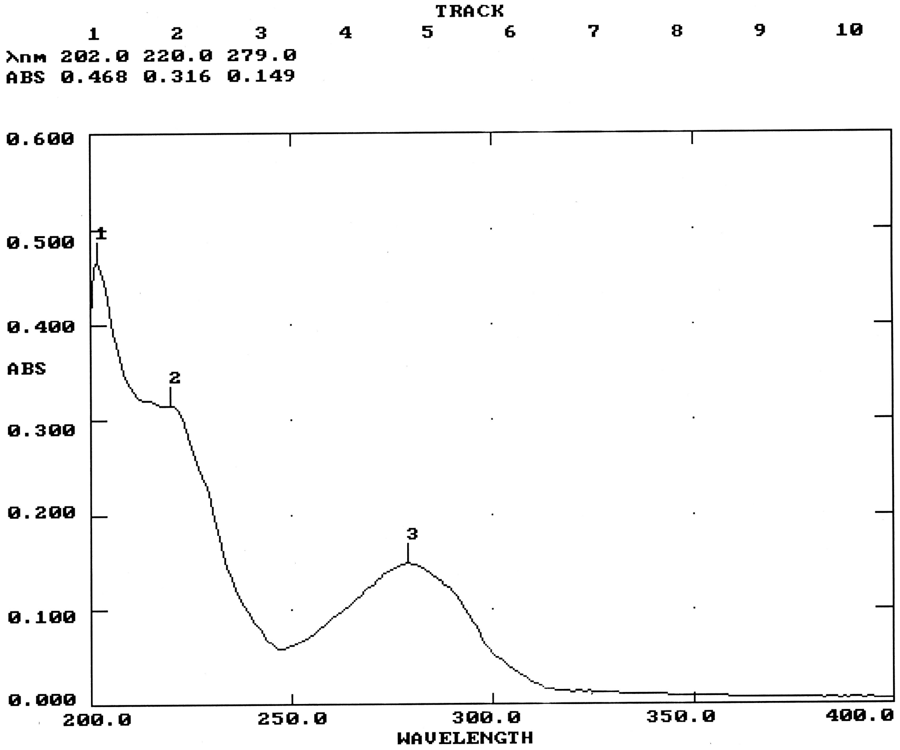

Figure S9. UV-Vis spectrum of compound **1**.

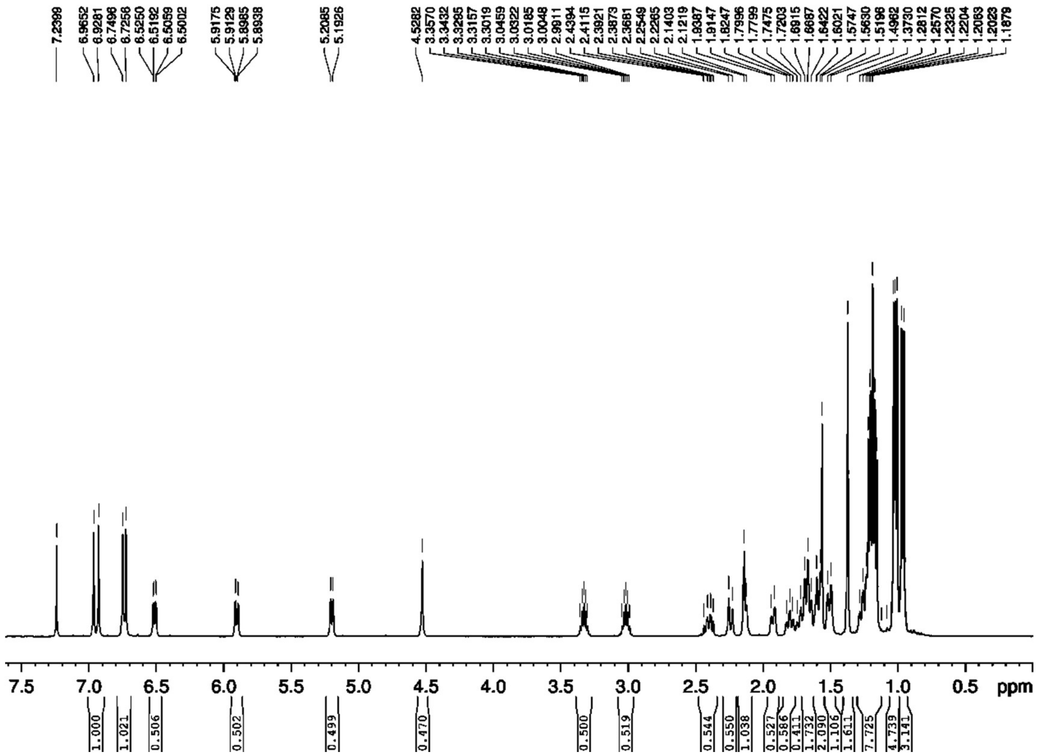

Figure S10. ^{1}H-NMR spectrum of compound **2** in $CDCl_3$.

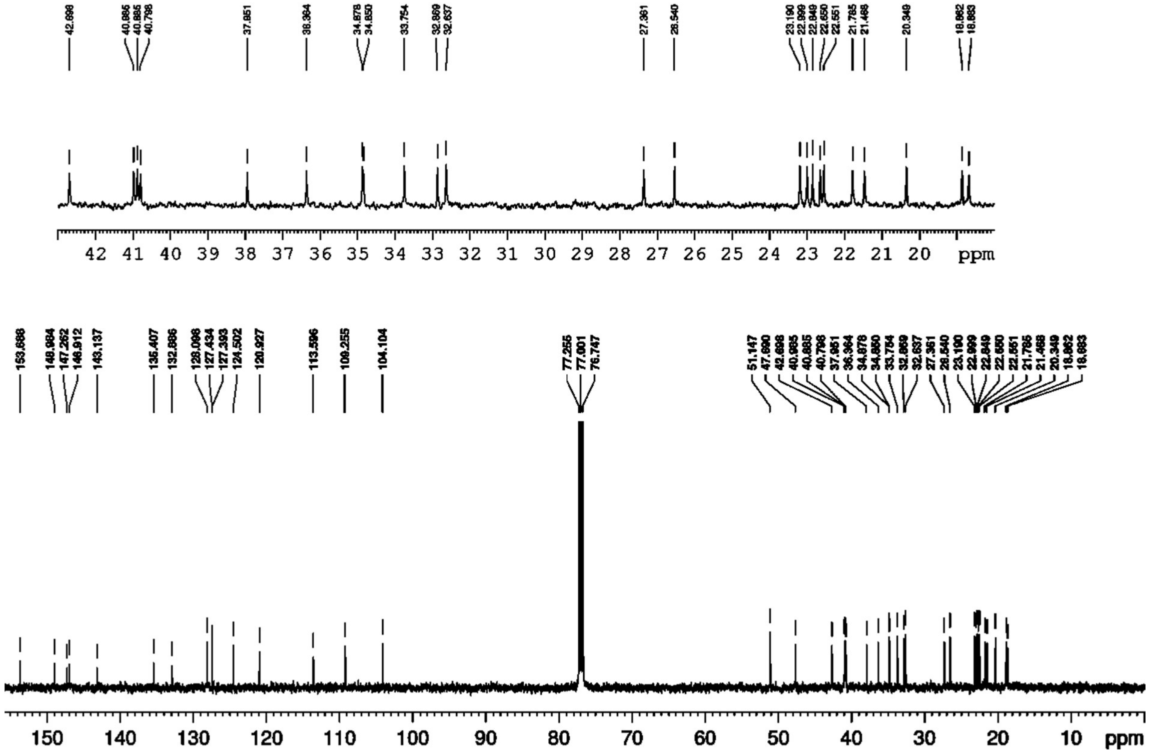

Figure S11. ^{13}C-NMR spectrum and DEPT of compound **2** in $CDCl_3$.

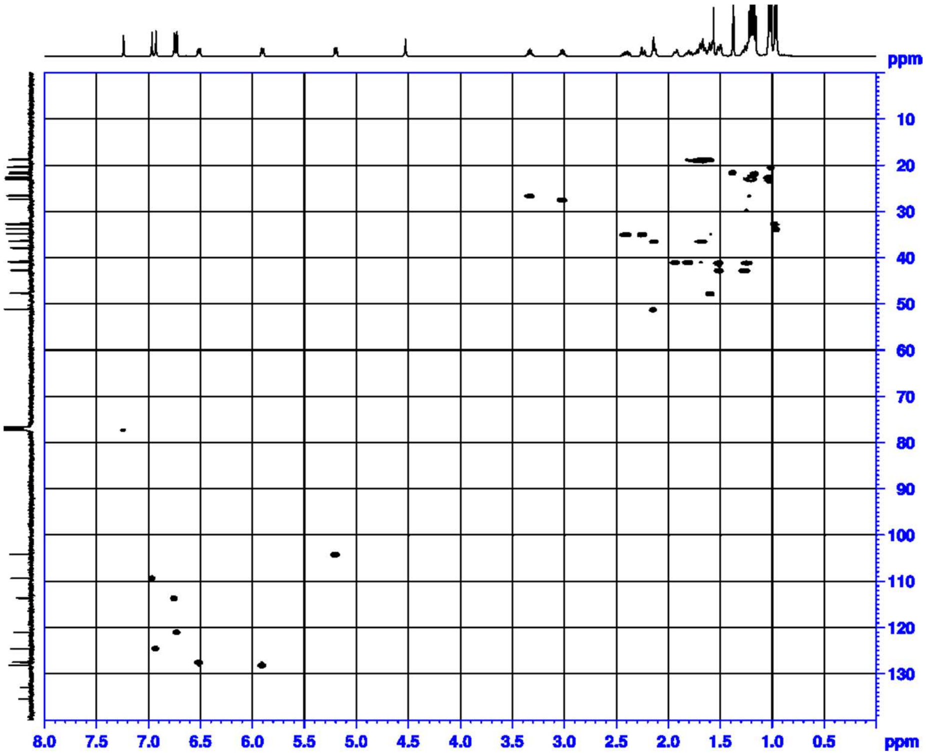

Figure S12. HMQC spectrum of compound **2** in $CDCl_3$.

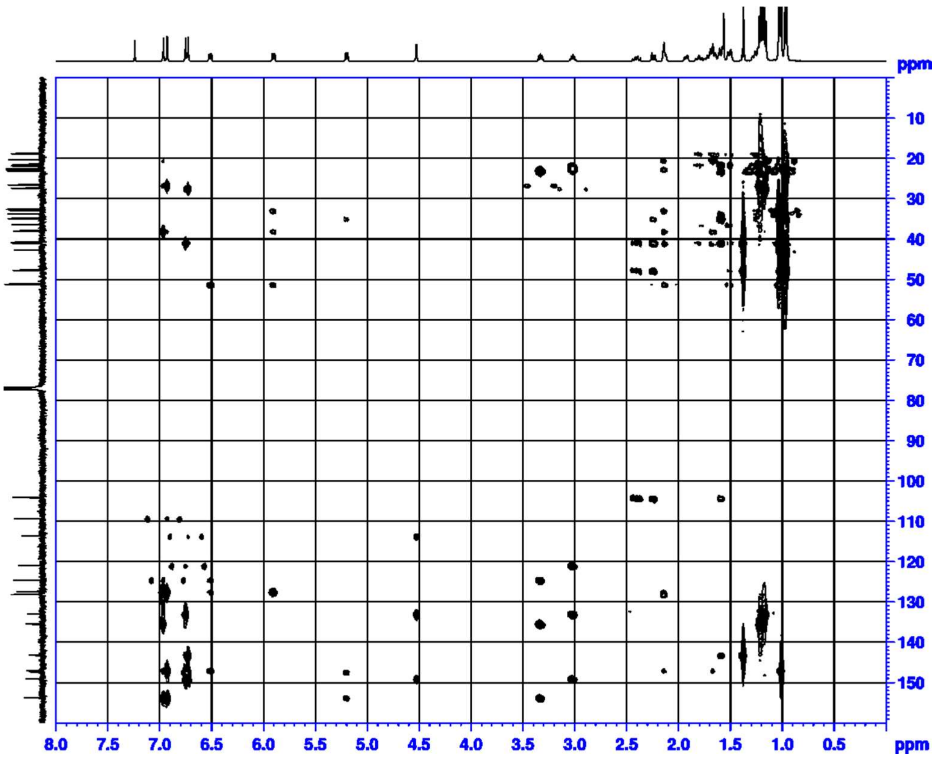

Figure S13. HMBC spectrum of compound **2** in $CDCl_3$.

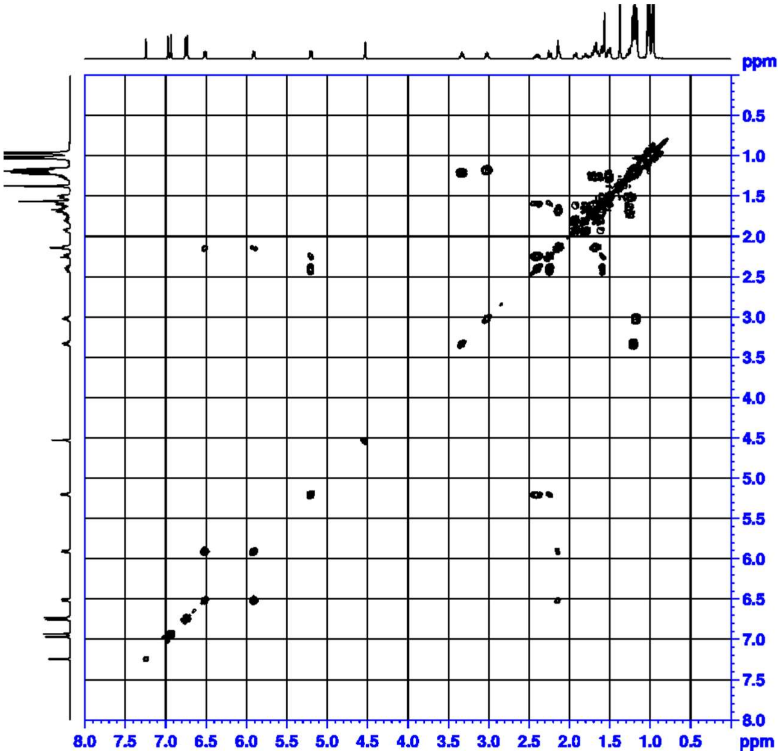

Figure S14. ^{1}H-^{1}H COSY spectrum of compound **2** in $CDCl_3$.

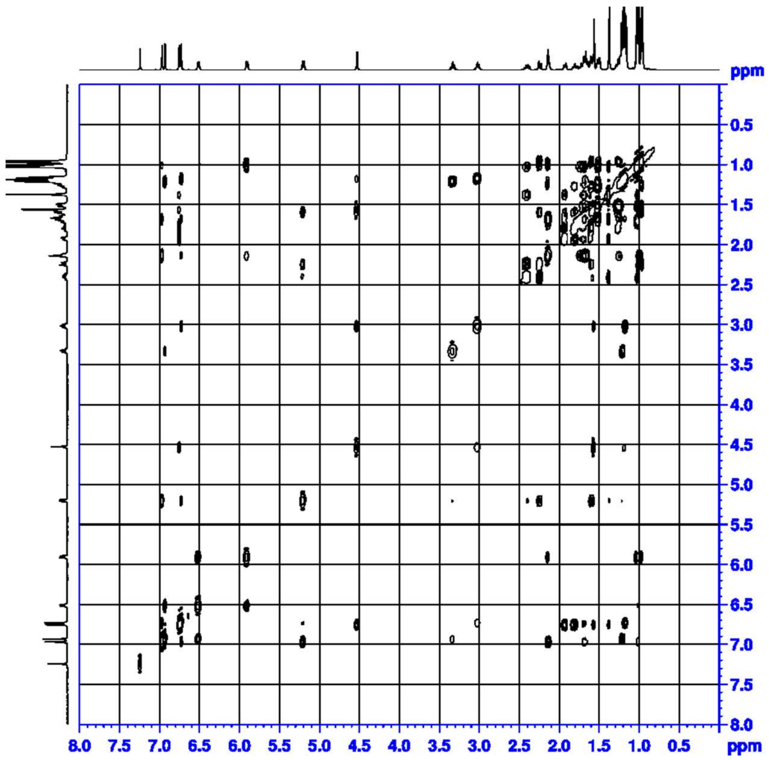

Figure S15. NOSEY spectrum of compound **2** in $CDCl_3$.

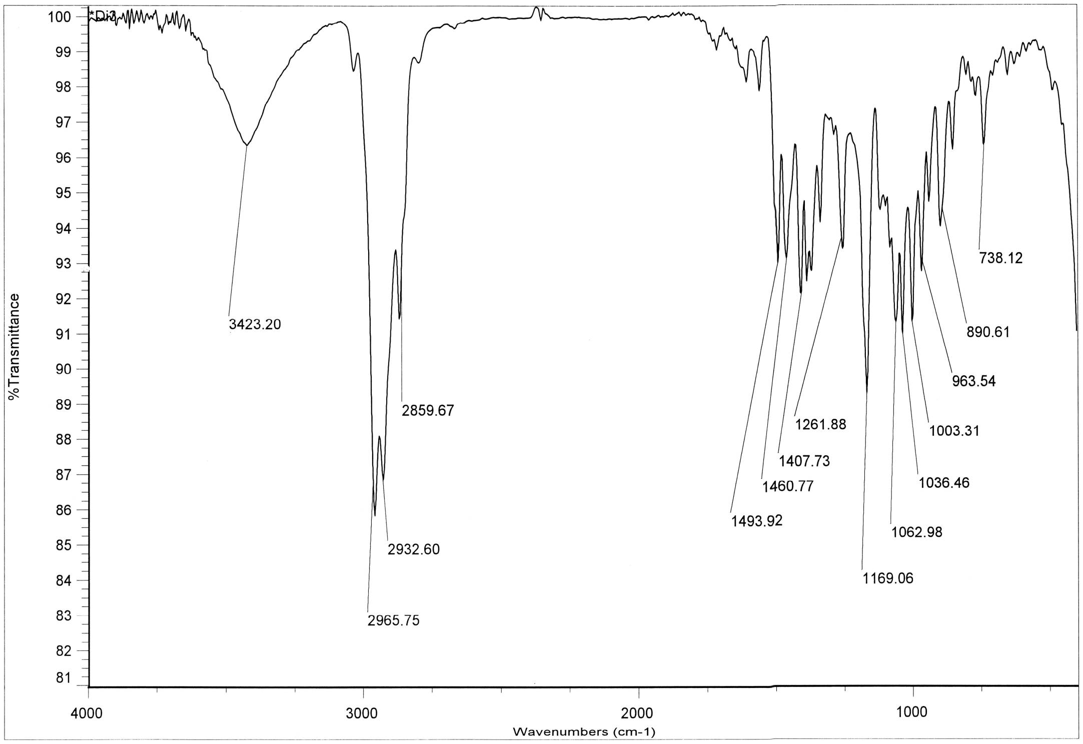

Figure S16. IR spectrum of compound **2**.

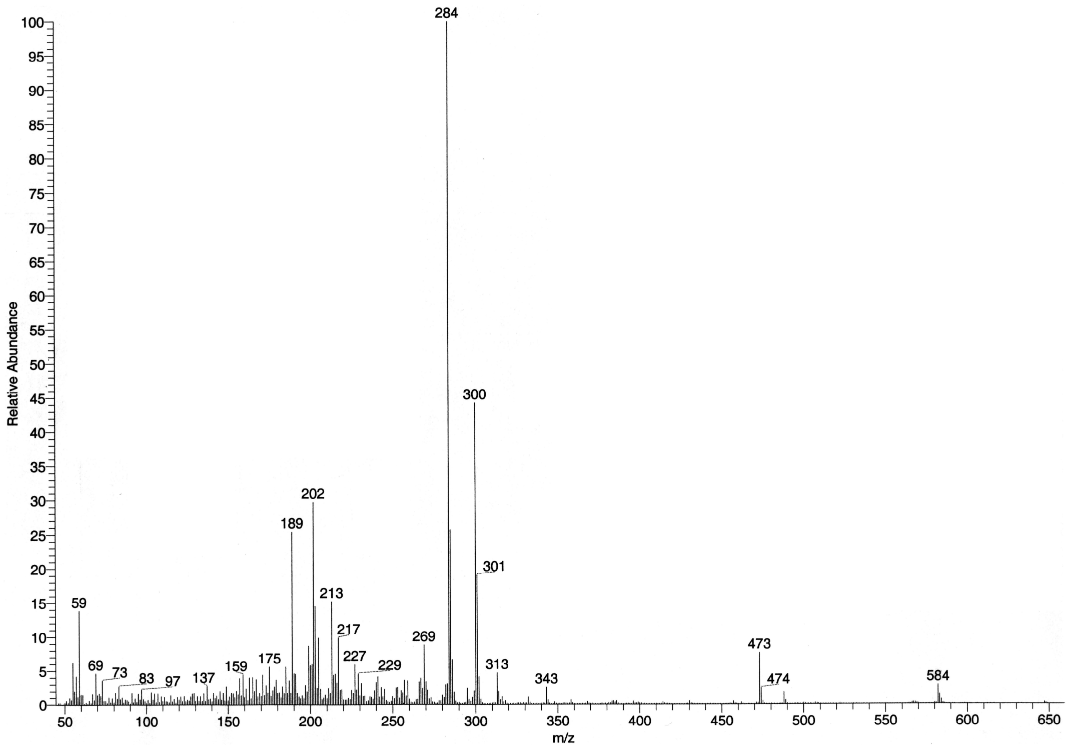

Figure S17. Mass spectrum of compound **2**.

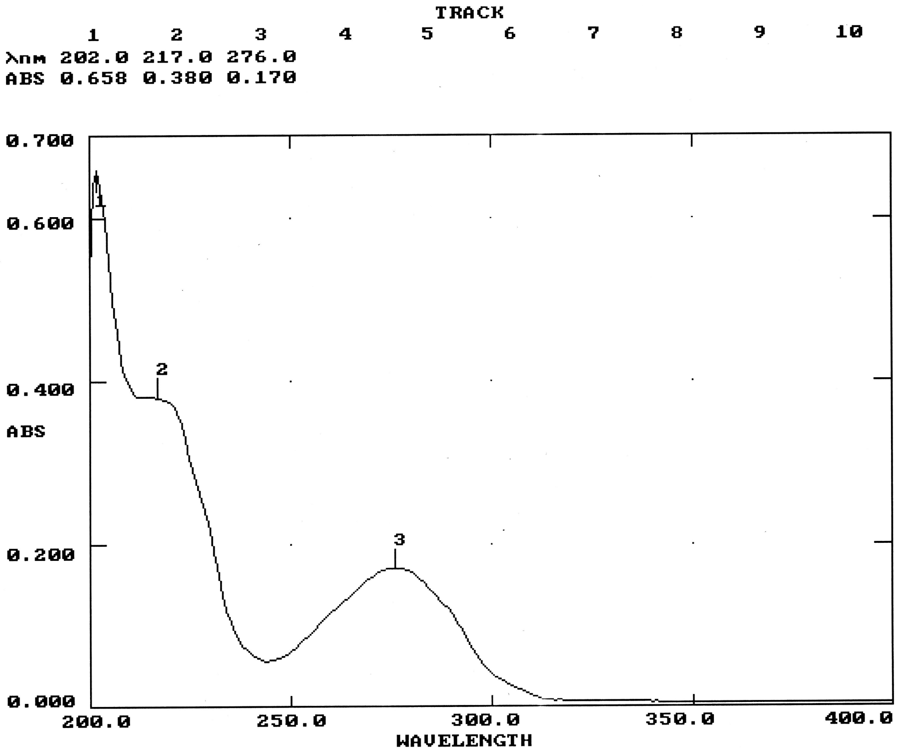

Figure S18. UV-Vis spectrum of compound **2**.

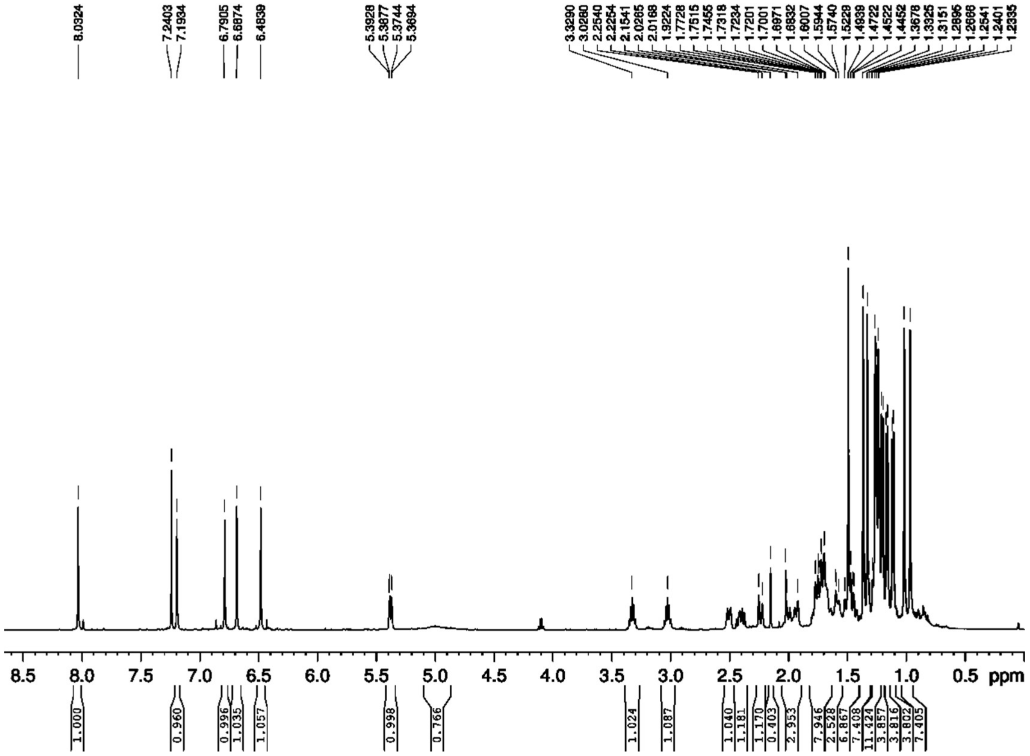

Figure S19. ^{1}H-NMR spectrum of compound **3** in $CDCl_3$.

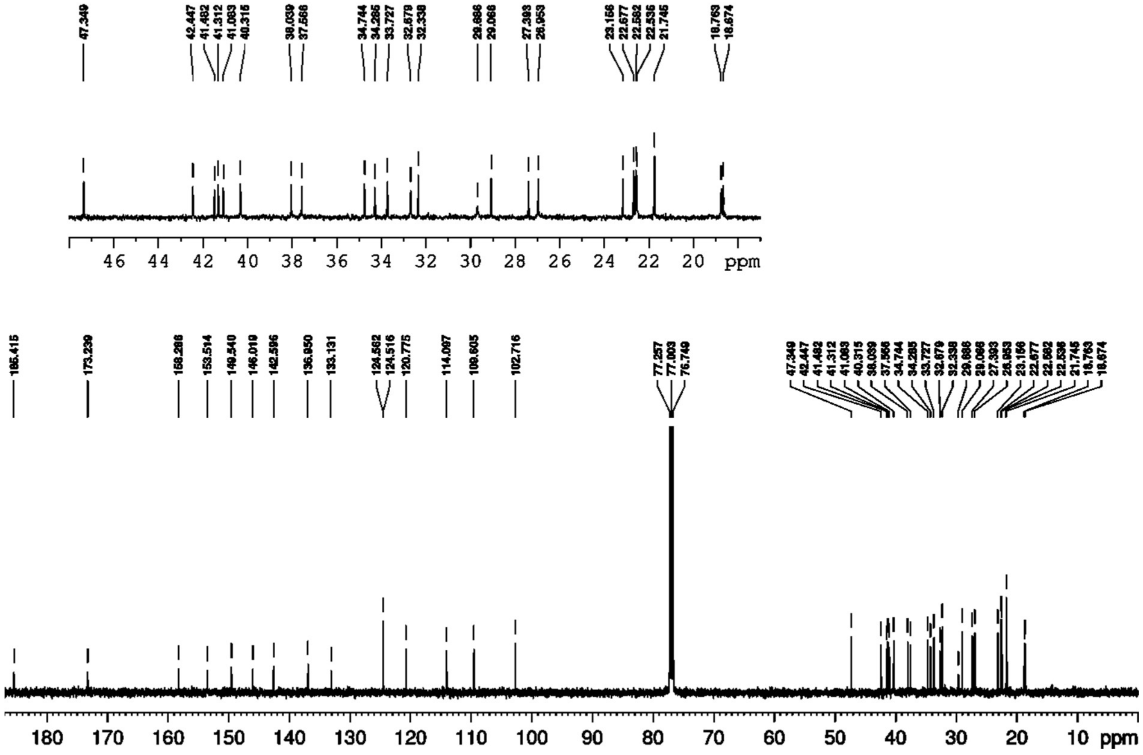

Figure S20. ^{13}C-NMR spectrum and DEPT of compound **3** in $CDCl_3$.

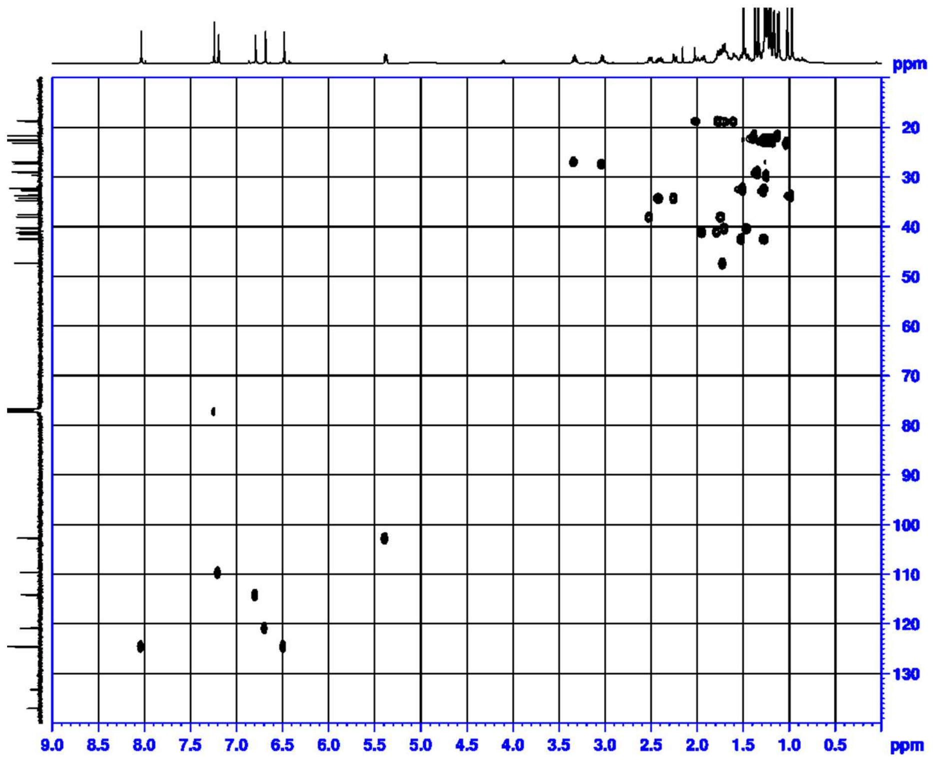

Figure S21. HMQC spectrum of compound **3** in $CDCl_3$.

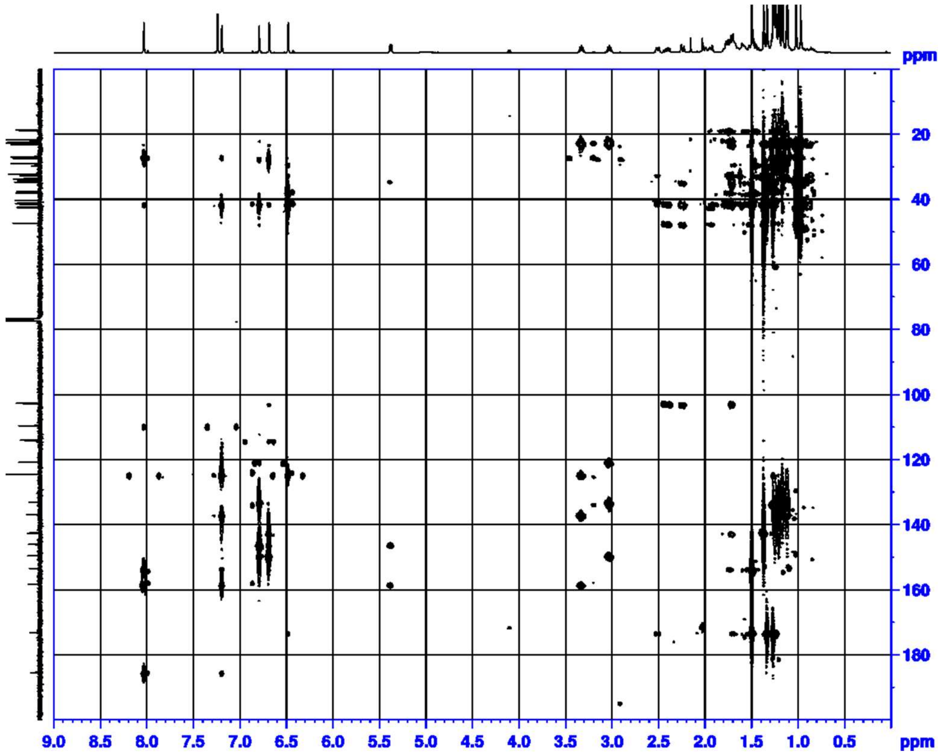

Figure S22. HMBC spectrum of compound **3** in $CDCl_3$.

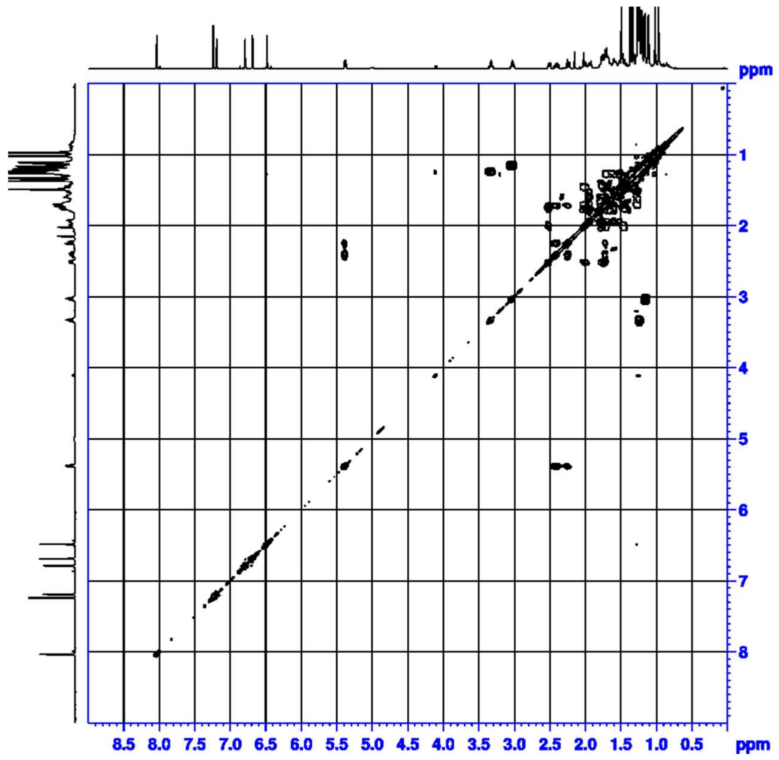

Figure S23. ^1H-^1H COSY spectrum of compound **3** in $CDCl_3$.

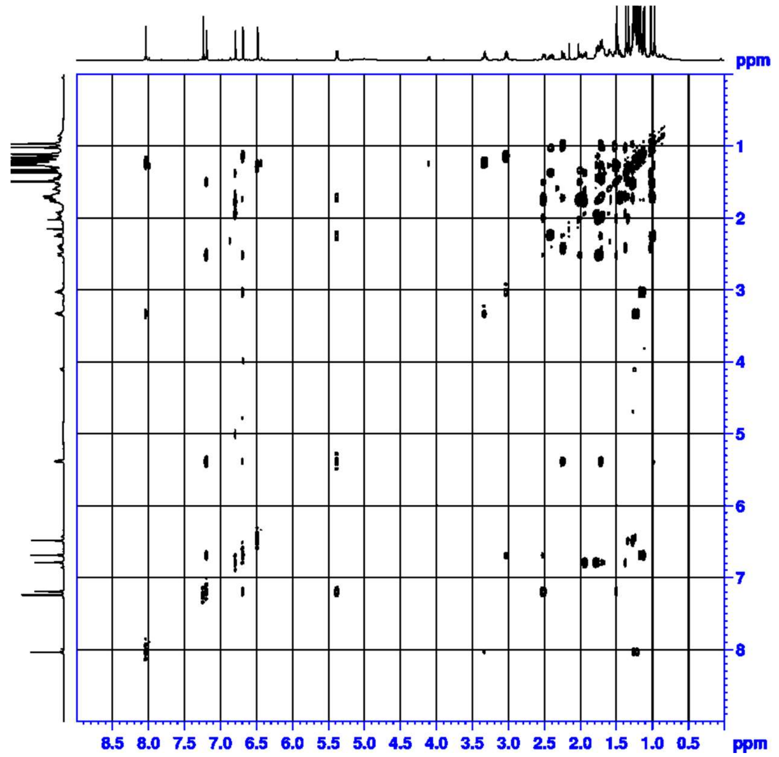

Figure S24. NOSEY spectrum of compound **3** in $CDCl_3$.

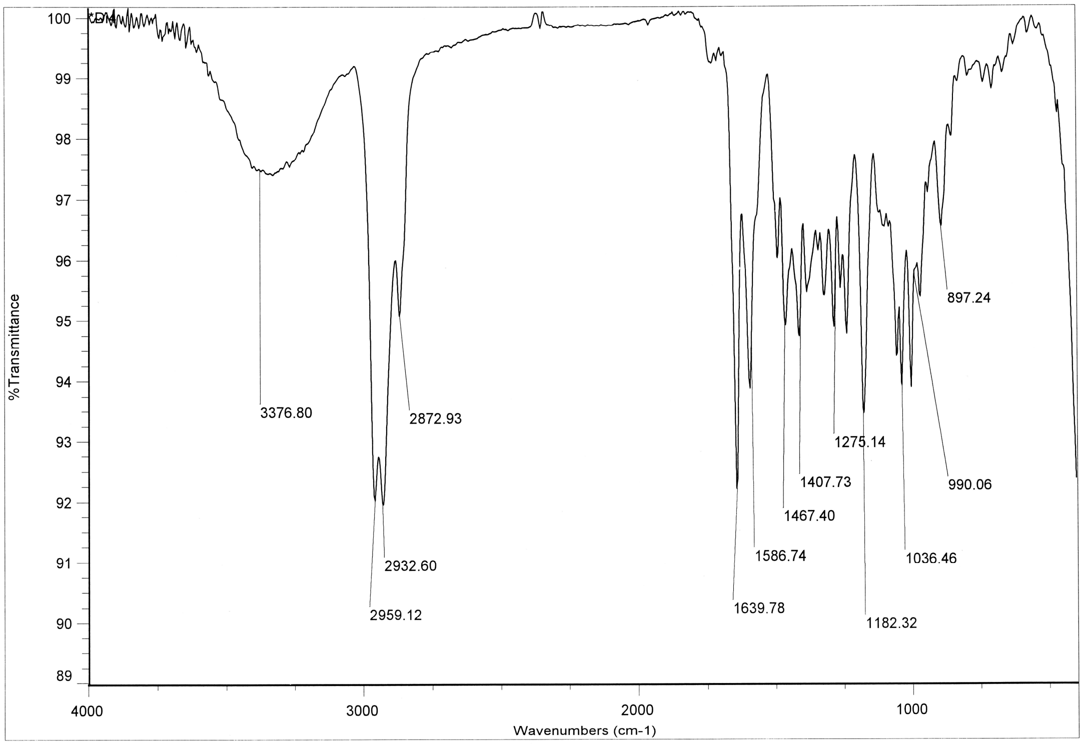

Figure S25. IR spectrum of compound **3**.

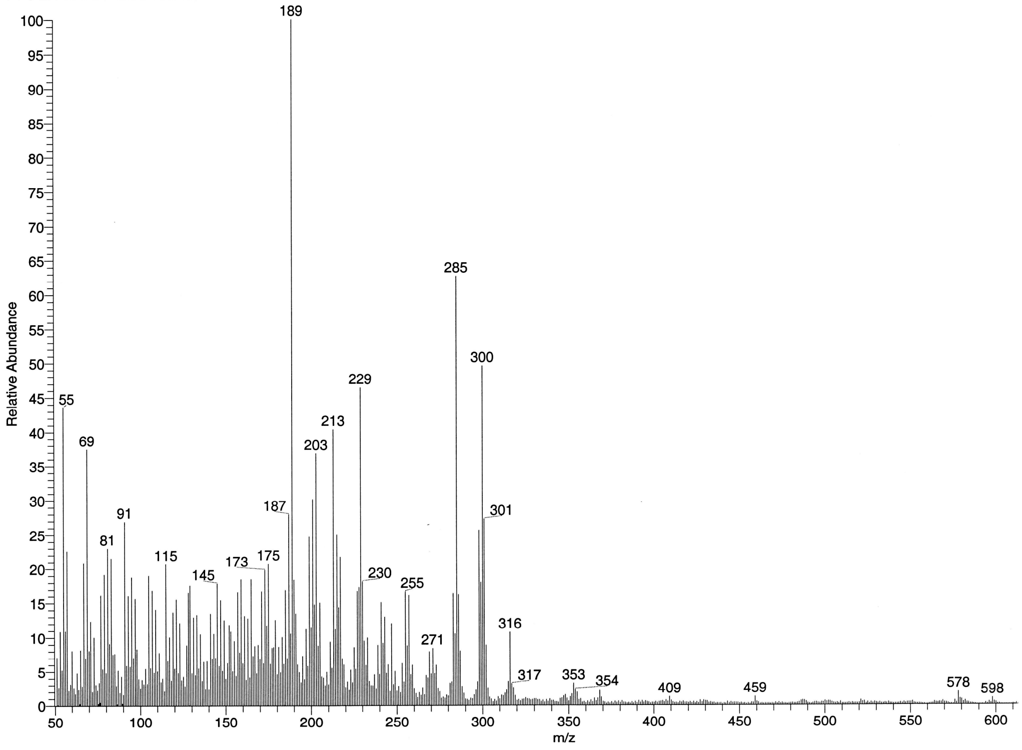

Figure S26. Mass spectrum of compound **3**.

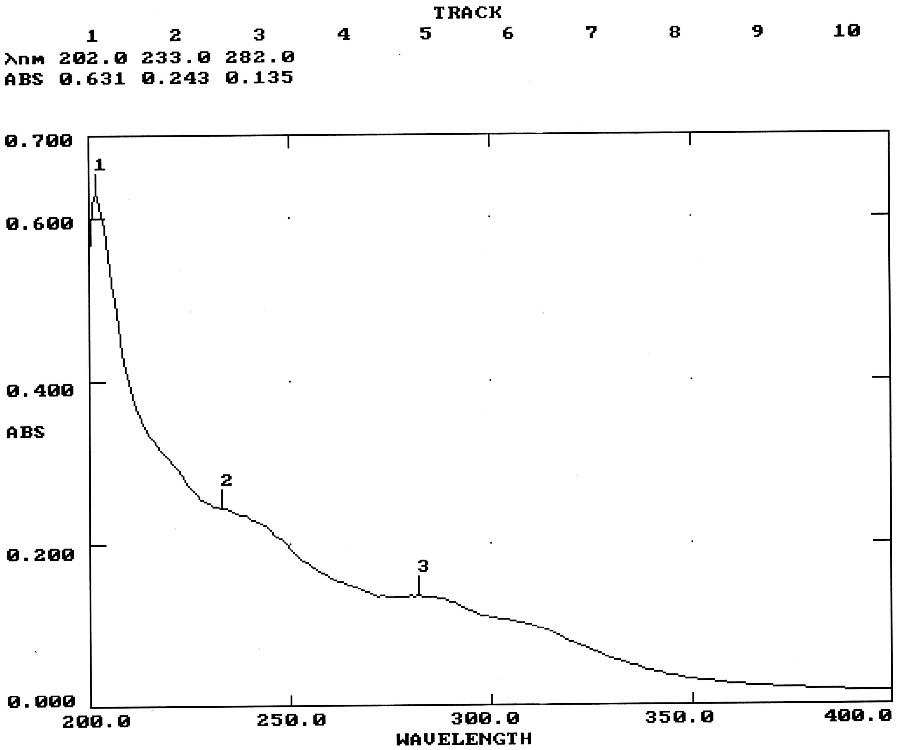

Figure S27. UV-Vis spectrum of compound **3**.

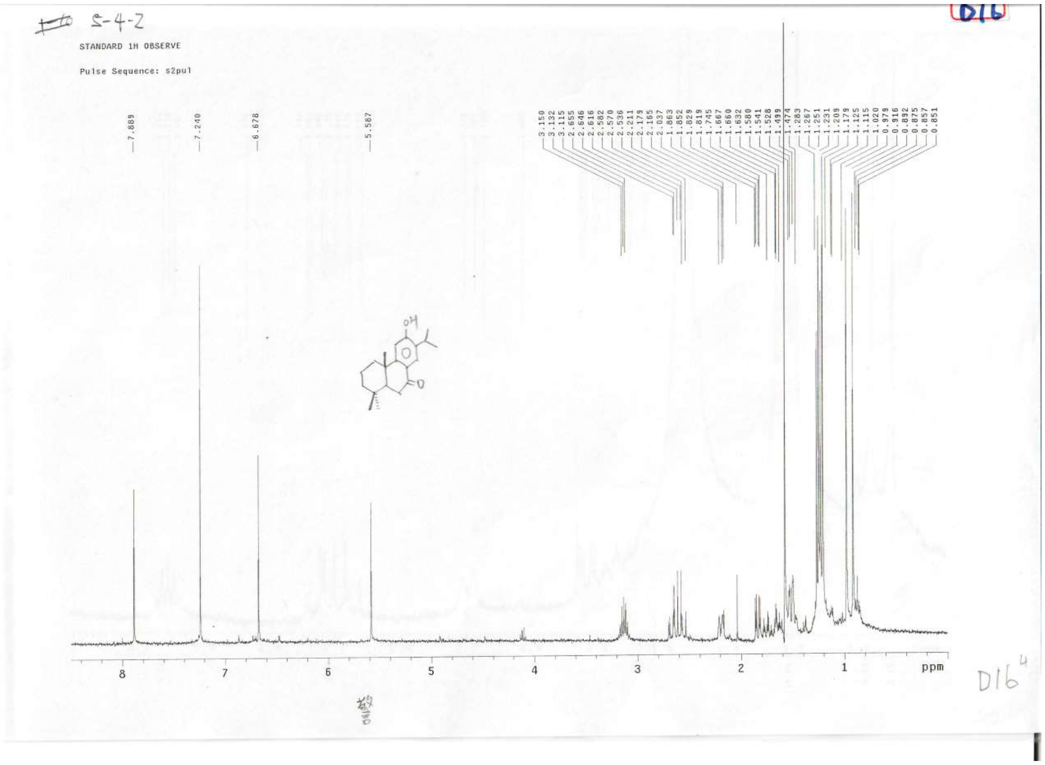

Figure S28. ^{1}H-NMR spectrum of compound **4** in $CDCl_3$.

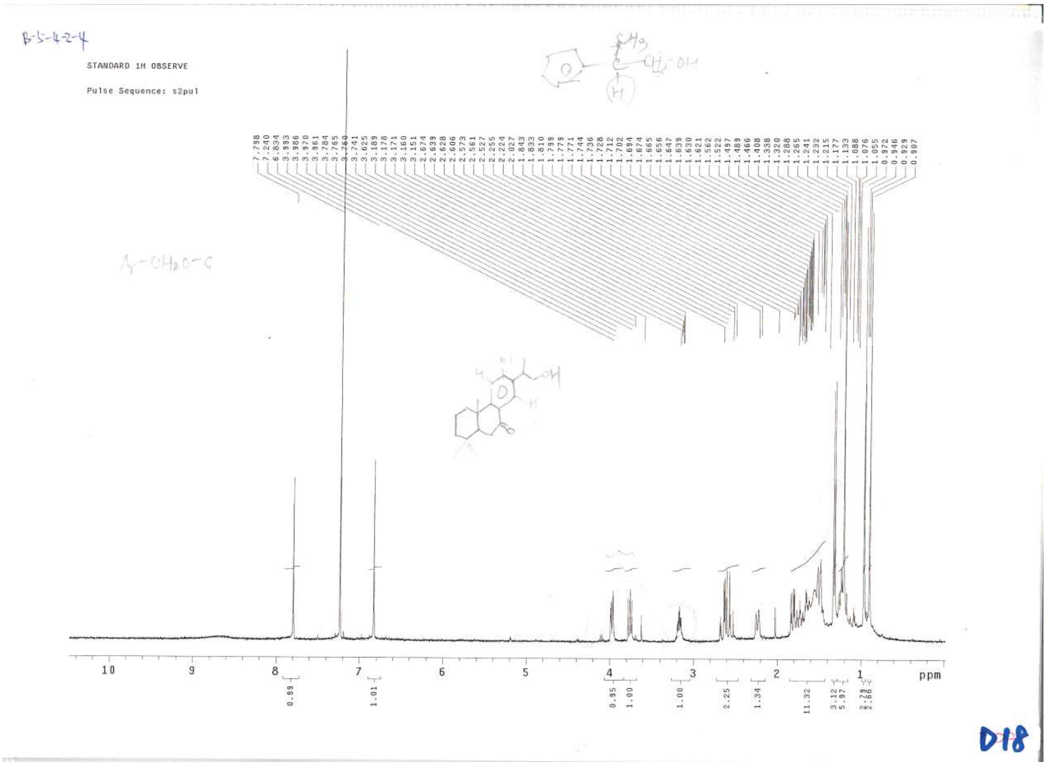

Figure S29. ^{1}H-NMR spectrum of compound **5** in $CDCl_3$.

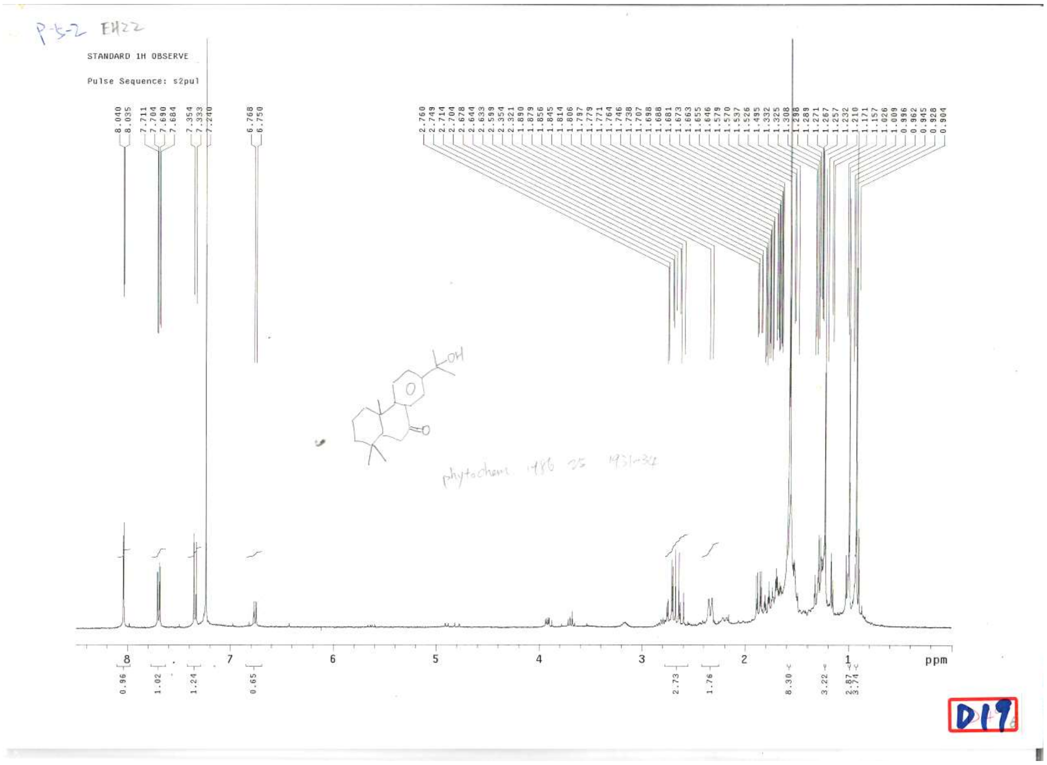

Figure S30. ^{1}H-NMR spectrum of compound **6** in $CDCl_3$.

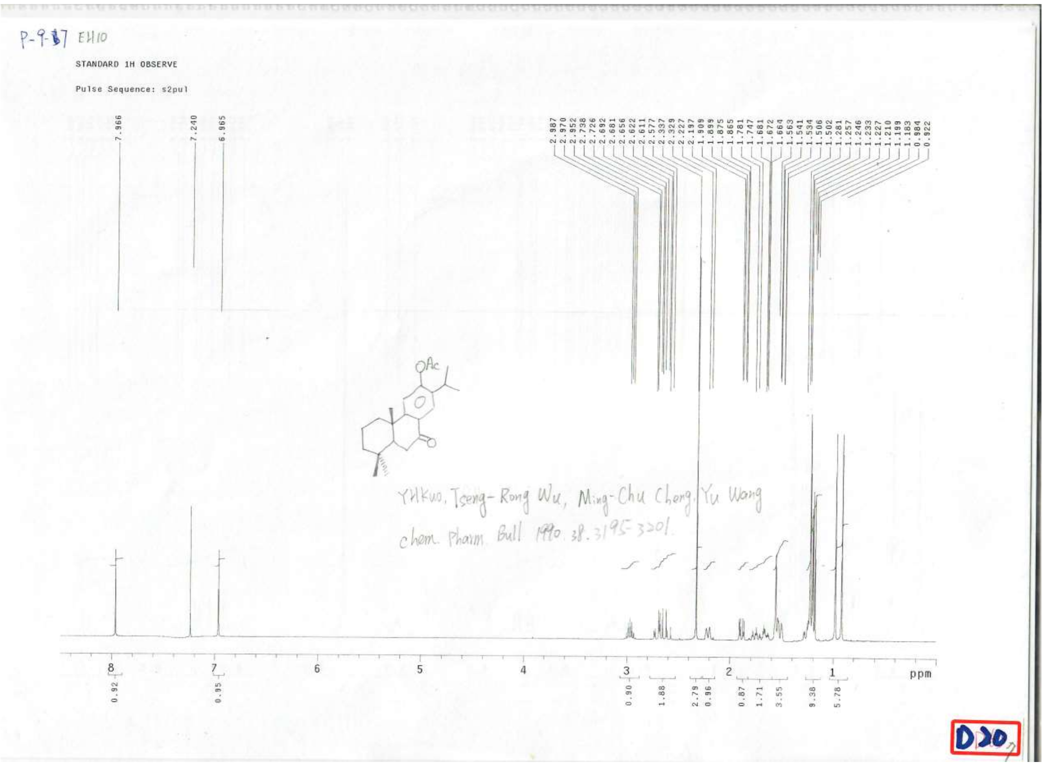

Figure S31. ^{1}H-NMR spectrum of compound **7** in $CDCl_3$.

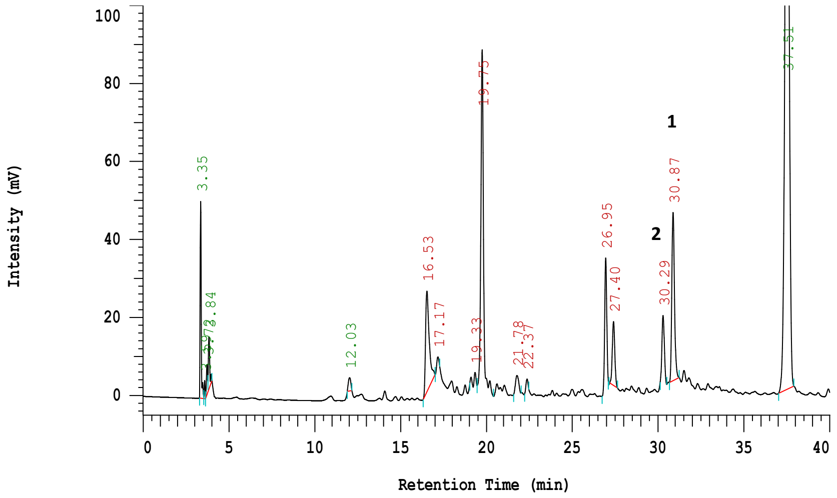

Figure S32. HPLC separation chromatogram of compounds **1** and **2**.
